# Different patterns of intrinsic functional connectivity at the default mode and attentional networks predict crystalized and fluid abilities in childhood

**DOI:** 10.1093/texcom/tgad015

**Published:** 2023-08-17

**Authors:** Diego Lombardo, Tobias Kaufmann

**Affiliations:** Department of Psychiatry and Psychotherapy, Tübingen Center for Mental Health, University of Tübingen, Calwerstraße 14, 72076 Tübingen, Germany; Department of Psychiatry and Psychotherapy, Tübingen Center for Mental Health, University of Tübingen, Calwerstraße 14, 72076 Tübingen, Germany; German Center for Mental Health (DZPG), Partner Site Tübingen, Calwerstraße 14, 72076 Tübingen, Germany; Norwegian Centre for Mental Disorders Research (NORMENT), Oslo University Hospital and Institute of Clinical Medicine, University of Oslo, Kirkeveien 166, 0450 Oslo, Norway

**Keywords:** crystallized abilities, fluid abilities, rs-fMRI functional connectivity, default mode network, attentional networks

## Abstract

Crystallized abilities are skills used to solve problems based on experience, while fluid abilities are linked to reasoning without evoke prior knowledge. To what extent crystallized and fluid abilities involve dissociated or overlapping neural systems is debatable. Due to often deployed small sample sizes or different study settings in prior work, the neural basis of crystallized and fluid abilities in childhood remains largely unknown. Here we analyzed within and between network connectivity patterns from resting-state functional MRI of 2707 children between 9 and 10 years from the ABCD study. We hypothesized that differences in functional connectivity at the default mode network (DMN), ventral, and dorsal attentional networks (VAN, DAN) explain differences in fluid and crystallized abilities. We found that stronger between-network connectivity of the DMN and VAN, DMN and DAN, and VAN and DAN predicted crystallized abilities. Within-network connectivity of the DAN predicted both crystallized and fluid abilities. Our findings reveal that crystallized abilities rely on the functional coupling between attentional networks and the DMN, whereas fluid abilities are associated with a focal connectivity configuration at the DAN. Our study provides new evidence into the neural basis of child intelligence and calls for future comparative research in adulthood during neuropsychiatric diseases.

## Introduction

Understand how neurocognitive networks compete for or cooperate for the computational resources of the brain to perform complex cognitive functions such as reasoning and problem-solving is among the primary research targets in cognitive neuroscience. In this sense, to characterize brain patterns of brain functional segregation and integration can help to understand how cognitive processes emerges in childhood (Deco et al. 2015; Schurz et al. 2020). However, given the small sample sizes in previous studies, or the incomplete socioeconomic characterization of the population, it remains poorly understood to which extent brain integration and segregation of resting-state networks relate to the emergence of reasoning in childhood (Tomasi and Volkow 2021). Studying the neural substrates and environmental factors determining general cognitive abilities during critical periods of neurodevelopment is important because it may help develop active strategies to enhance children’s attention and learning.

The degree to which the default mode network (DMN) is relevant to the emergence of complex cognitive functions is still debated. Evidence suggests that the DMN is one of the networks with more structural and functional changes during child neurodevelopment (Menon 2013). But even though the DMN has been proposed as an important network to the emergence of complex cognition (Smallwood et al. 2021), it remains poorly understood how the DMN is functionally associated with task-positive networks relevant to allocate attentional resources (Fox et al. 2006; Corbetta et al. 2008) needed for complex cognition (Ramchandran et al. 2019), such as dorsal attention (DAN) and ventral attention networks (VAN). Nevertheless, a large body of evidence supports that functional connectivity of the DAN and VAN is essential to allocate cognitive resources to process complex conceptual thoughts in healthy adults (Dhamala et al. 2021). But furthermore, recent evidence suggests that functional connectivity between DMN and DAN relates to cognition during a task in adults (Dixon et al. 2017) and that the established functional anti-correlation between DMN and DAN is reduced with aging (Spreng et al. 2016). In the same line, DMN DAN anti-correlation has been shown to predict attention problems in children (Owens et al. 2020). In the same line, recent evidence suggests that a balanced level between segregation and integration is needed for effective behavior and cognition (Wang et al. 2021; Fransson and Strindberg 2023). Importantly, it remains poorly understood how the system-level segregation balance between task-negative networks as the DMN, and attentional networks, relates to complex mental processes in childhood that depend on an adequate attention span such as intelligence.

It has been shown that socioeconomic factors can change brain functional networks (Deary et al. 2010), and shape human cognitive abilities (Hackman and Farah 2009). However, given the complex matrix of interacting factors that determine intelligence, such as sex (Tomasi and Volkow 2023), income (Tomasi and Volkow 2021), parental education (Rindermann and Ceci 2018), and psychosocial adversity (Blair 2006), it remains challenging to isolate and characterize the actual weight of every dimension affecting problem-solving and reasoning abilities. Since human intelligence is a complex construct, it has been proposed that cognitive skills can be dissociated into crystallized abilities (Cattell 1963), more dependent on previous knowledge and fluid abilities or problem-solving skills based on abstract reasoning (Horn 1973). Importantly, even though the functional and brain structural basis of fluid and crystallized capabilities has been studied with several paradigms in adults (Geake and Hansen 2010; Román et al. 2014; Dhamala et al. 2021), the neural substrate underlying fluid and crystallized abilities in childhood, and relative importance of neural and non-neural factors, remain poorly understood.

In the current study, we aimed to understand the contribution of large-scale coupling of the DMN and attentional networks to the emergence of fluid and crystallized abilities in childhood. For this, we analyzed patterns of resting-state functional connectivity from a large population of children relucted in the multicentric ABCD study to understand whether segregation and integration between DMN and attentional networks predict fluid and crystallized abilities in childhood. Further, we sought as a secondary aim to map the relative importance of biological network factors compared with socioeconomic factors determining fluid and crystalized cognition.

## Material and methods

### Procedures

The Adolescent Brain Cognitive Development (ABCD) study is an extensive ongoing observational, longitudinal, and multicentric study with a sample recruited from 21 research sites across the US. The study aims to understand variations in normal cognition and behavior in adolescence, a critical period of neurodevelopment (Volkow et al. 2018), along with variations of typical cognitive trajectories by external factors. ABCD adopted epidemiological principles for recruiting (Garavan et al. 2018) to ensure demographic variations similar to in general demographic features of the US population between 9 and 10 years old. Consent was obtained from parents and assent from participating children. The appropriate institutional review boards approved the protocols and approved the ABCD study.

### Sample

In this study, we use data from the baseline visit. Children were included if they had at least one resting-state fMRI run that passed the first quality control (QC) checks. Assessment details can be found in (Hagler Jr et al. 2019). To ensure quality, two trained technicians performed a visual inspection reviewing the severity of five categories of artifacts for cortical surface reconstruction. Framewise displacement (FD) greater than 0.2 mm led to exclusion. MRI sessions with fewer than 5 min of good scan time (i.e. 375 remaining volumes) after censoring for motion (<0.2 mm per volume) were also removed.

**Fig. 1:**
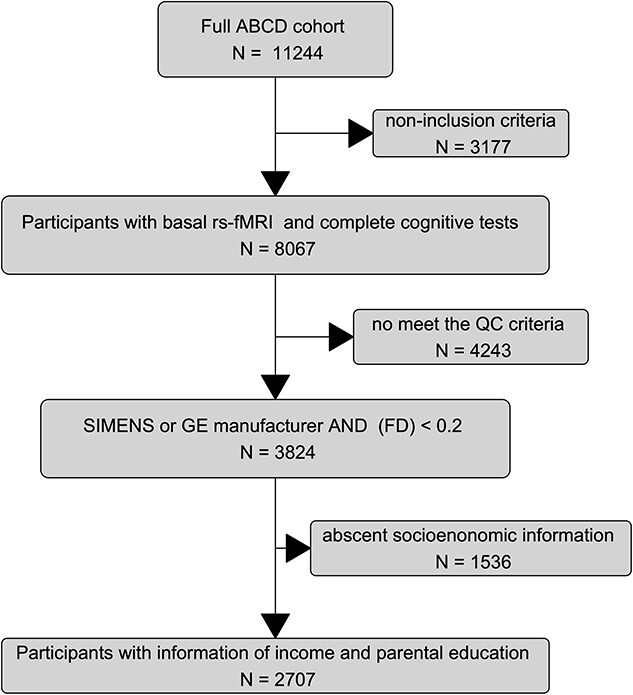
Flowchart detailing the procedure implemented to select participant data in this study. (FD) denotes average head framewise displacement, and (QC) quality control.

In addition, study-specific criteria were applied. As summarized in the flowchart in Fig. 1, participants were included if they had a) rs-fMRI, complete cognitive evaluations, and socio-demographic information, b) Quality control of the MRI based on Framewise displacement (FD) average < 0.20 mm as suggested by (Power et al. 2014) c) Their scan was performed with SIEMENS or GE Medical system manufacturers (see fMRI section). The final features of the cohort is described in Table S1, N = 2,707 participants with 1405 girls, and 1302 boys.

### Cognitive assessment and demographic data

Every participant was assessed with several cognitive tests from the NIH Toolbox (Weintraub et al. 2013), evaluating verbal and visual episodic memory, attention, language, working memory, executive functions, and fluid and crystallized abilities. The NIH Toolbox Cognition Battery (NIHTB-CB) composite scores on fluid, and crystalized intelligence has previously been validated as an accurate measure of intelligence (Heaton et al. 2014). Fluid intelligence represents the ability to use new information to abstract reasoning independently of previous experience. On the other hand, crystallized skills are described as close to verbal skills dependent on accumulated experience. Indeed, crystallized and fluid abilities capture individual variability in brain structure that can’t be caught with a single *g* factor of intelligence (Simpson-Kent et al. 2020). In this study, we focused on four computerized tests. First, we analyzed neuropsychological data from the NIH Toolbox cognitive assessments, which evaluate neurological and behavioral functions. The toolbox consists of several tasks, including fluid and crystalized intelligence computer-based assessments that have previously been shown to have excellent test–retest reliability in childhood (Akshoomoff et al. 2013). To have a more extensive evaluation of fluid and crystallized abilities, we took into account cognitive tests, essential for the emergence of crystalized cognition, such as the picture Vocabulary Test and fluid capabilities, and List Sorting test assessing executive functions and working memory central for fluid abilities (Scott et al. 2019). These tests were administered by trained research staff using a laptop. Other evaluations assessed multiple cognitive domains such as attention, executive functions, processing speed, episodic memory, reading, overall cognition, and visuospatial abilities. In a broad sense, we quantified crystallized and fluid cognition, giving special weight to working memory on fluid intelligence and verbal skills in crystallized abilities as we justified before. For this, we build a first set of scores as an average from A) cognitive evaluations of fluid intelligence raw scores (“nihtbx_fluidcomp_uncorrected”) and short List working memory (“nihtbx_list_uncorrected”) is our composite score in fluid abilities, and B) Crystallized intelligence raw score (“nihtbx_cryst_uncorrected”) and picture vocabulary test (“nihtbx_picvocab_uncorrected”) is our composite score in crystilized abilities. All measures of crystallized and fluid cognition, either isolated or averaged together, have shown excellent test–retest reliability in child (Akshoomoff et al. 2013; Luciana et al. 2018).

Data were obtained from databases “ABCD Youth NIH TBS summary Scores” short name: “abcd_tbs01.” The demographic information was obtained in the same query from the ABCD Longitudinal Parent Demographics Survey, release 2.0; short file name “abcd_lpds01.” Categorical covariates were transformed into factors using R version 4 2.2., tables were built based on raw scores on ABCD Youth NIH TB Summary Scores. Details about the processing and codes used for the analysis are shared via GitHub: https://github.com/diego1977-code/diego1977-code.

### fMRI data acquisition, preprocessing, and processing

This study analyzed resting state functional connectivity of a final cohort of N = 2707 participants, preprocessed as part of the ABCD 2.0 release. Imaging was performed across 21 sites within the United States, with harmonized protocols across the acquisition devices (Siemens, GE, and Philps). On December 2, 2019, the ABCD study publicly announced the incorrect post-processing of resting-state and task-evoked fMRI data indicating incorrectly specified fieldmaps from Philips scanners (Nielson et al. 2018). Therefore, in our study, we have only considered resting-state fMRI sessions from Siemens Prisma and GE 3 T MRI devices. Each fMRI acquisition block includes fieldmap scans for B0 distortion correction, which is needed for further fMRI processing. Specific details on image acquisition can be found in (Casey et al. 2018).

Resting-state BOLD signal was processed following the ABCD Data Release 2.0 processing pipeline, launched in March 2019. A description of the procedures for processing and processing rs-fMRI is detailed in (Hagler Jr et al. 2019). Denoising regressors comprise signal and movement variables. Signal regressors were mean time series for white matter, CSF, and the global signal. Standard preprocessing performed by DBP included applying a respiratory motion filter and motion variables comprising 3 translational and 3 rotational regressors. Then, the BOLD time series were filtered between 0.008 and 0.09 Hz by using a 2^nd^ order filter. Finally, the construction of parcellated time series was projected to the Gordon atlas template with 422 parcells (Gordon et al. 2016). Pearson’s correlation values for each pair of ROIs were Fisher transformed to z-statistics and averaged within or between-network of interest. The average correlation within a network or within-network connectivity was calculated as the average correlation for each unique pairwise combination of ROIs within an rs-network, and between-network connectivity was calculated by averaging the pairwise combination of ROIs in the first network with the ROIs of the second network. The data was packed in the resting-state fMRI file named “abcd_betnet02.” Because differences between MRI manufacturers had been identified, MRI manufacturer was included as a covariate in the statistical models, as recommended by (Hagler et al. 2019); the MRI manufacturer information was obtained from the database “abcd_mri01.

### Divergent functional coupling between DMN and attentional networks

**Fig. 2:**
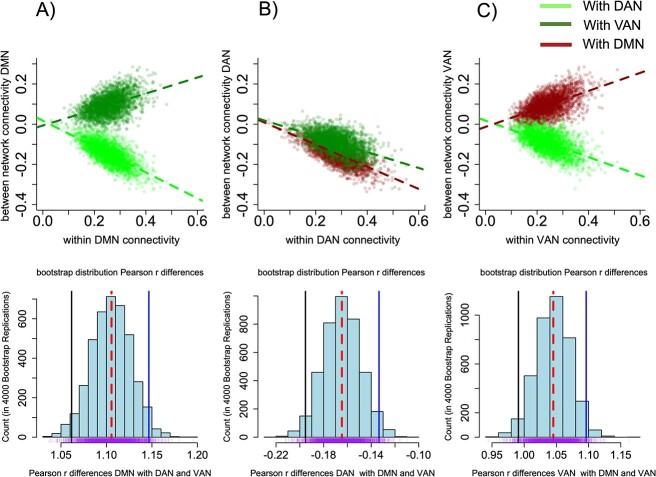
Functional coupling between the DMN and attentional networks reveals the anticorrelated configuration of the DAN in children. The upper figure in panel A shows the scatter plots for the correlation between within-network connectivity of the DMN with between-network connectivity between DMN dorsal attention (DAN) and ventral attention networks (VAN) in the whole cohort (N = 2707). The lower figure in panel A shows the bootstrap distribution of Pearson’s correlation differences plotted in the upper figure of panel A. The lower histogram in panel B shows the bootstrap distribution for the differences in Pearson’s correlation coefficients between DAN within-network connectivity and DAN between-network connectivity with DMN and VAN (purple lines denote individual bootstrap samples). As observed in the scatter plots of the upper figure panel in B, both distributions are superimposed (both anticorrelated with DAN dashed regression lines red and green). This pattern denotes that within-network connectivity of the DAN expresses a symmetric anticorrelation with VAN and DMN connectivity. The histograms below show that a near one value of the mean of Pearson’s r correlation differences between within-network connectivity of a given network and between network connectivity of this network and the other two networks (mean in red dashed line) denotes an asymmetric coupling (anticorrelated with one network and correlated with the other one), the pattern observed for in panels A and C. Near to zero value of Pearson’s r correlation differences denotes a symmetric coupling between the seed network and the large-scale coupling with the other two networks, as is the case for the DAN in panel B. These differences are evident in the confidence intervals of the bootstrap distributions in the histograms (2.5% percentile in solid black, and blue lines 97.5% percentile). Note that the distributions in lower histograms in panels A and C graphs almost overlap around 1. The confidence intervals for the correlation differences are near to zero when DAN is the seed network (lower figure in panel B), which is then times smaller than when the seed network is the DMN or VAN. The confidence intervals for the histograms of the bootstrap distribution of the DMN in panel A (Ci 1.061656: 1.146614, mean = 1.105563) for the histogram of the VAN in panel C (Ci 0.9910644, 1.096345, mean 1.045198) while for the DAN (Ci 0.195289, − 0.1334365, mean = −0.1646476. This evidence shows that the within-network connectivity of DAN is symmetrically anticorrelated with DAN and VAN connectivity. On the other hand, the DMN and DAN express asymmetric coupling with the other two resting-state networks.

To compare the degree of coherence between within-network connectivity at the DMN and the between-network connectivity with DAN VAN, we calculated the Pearson’s correlations coefficients between within-network and between-network connectivity of DMN, DAN, and VAN in Fig. 2. Using the R package “bootcorci” we compared correlation’s differences calculated by bootstrapping Pearson’s r by 4,000 bootstrap repetitions (histograms of the bootstrap distributions in lower graphs of Fig. 2).

### Statistical models

We calculated multivariate linear regression models to test for association with fluid and crystallized cognition of within-network connectivity of the DMN DAN VAN and between network connectivity between these networks. As covariates, we included age, sex, household income, parent’s education, and MRI manufacturer. To account for batch effects and the nested covariance structure of the ABCD dataset, site “site_id” was added as a covariate to the linear regression models. Because head motion remains a confound in the rs-fMRI analysis in the ABCD study (Cosgrove et al. 2022), we correlated average framewise displacement (FD) with all our behavioral and functional connectivity measures. Even though there is only an small negative correlations between FD and fluid intelligence (Fig. S1), we added FD as a covariate to the statistical linear models. All analyses were performed in R version 4.2.2 to linear regression model’s cross-validation using repeated K-folds (10 folds, 5 repetitions) using the package “caret.” As recommended by (Scheinost et al. 2019), cross-validation of predictive models with 5 repetitions and low values of k-fold (5- or 10-fold cross-validation) is a good strategy for cross-validation in large samples, while a higher number of folds would lead to overfitting (Varoquaux et al. 2017). Prediction accuracy performance was estimated using training and test samples (see features of these samples in Table S3), for every model we quantified the Spearman correlation rho between observed and predicted values of crystallized and fluid cognition, R^2^ or the coefficient of determination, was computed for every model. To estimate the amount of variance of cognitive performance explained by single factors of within and between-network functional connectivity at the DMN DAN VAN and avoid effects of nuisance variables that might increase the odds of overfitting, we re-calculated the linear regression models, including as a single predictor resting-state functional connectivity FC, without adding any other covariate (see Table 1). The coefficients of the regression with only the network FC factor were also calculated by bootstrap distribution of β coefficients in random half-split samples (see features of these random samples in Table S4). Given that a linear regression model with multiple predictors might lead to the risk of overfitting (Babyak 2004) and more simplistic models with few predictors to underfitting, we also performed LASSO regression models since penalization and shrinkage offer a good balance between adding control variables to the model and penalizing potential nuisance factors. Importantly, LASSO regression models were also calculated to assess the relative importance of brain and non-brain factors explaining crystallized and fluid cognition since the penalization performed by LASSO gives a more accurate estimation of the β coefficients (Melkumova and Shatskikh 2017). The LASSO regression models had the same covariates as the abovementioned multivariate linear regression models. As described above, the outcomes were the two *average scores:* in *fluid abilities* (raw score on fluid intelligence with short List working memory) for one side, and *crystallized abilities* (raw score on crystallized intelligence with picture vocabulary test).

**Table 1:** The table report the results of the predictive models having as an outcome composite score on crystallized and fluid abilities.

	*Crystalized cognition*					
	Repeated k-folds Only fMRI rs-FC		Repeated k- folds All covariates		**Lasso at lambda 1 se**	
	**R** ^ **2** ^	**rho**	**R** ^ **2** ^	**rho**	**rho (R** ^**2**^)	**β**
DMN	0.002859	0.05977801	0.20	0.413^*^^*^^*^	0.46 (0.212)	2.3
DMN DAN	0.007929	0.09437769 ^*^^*^	0.2441^*^^*^	0.412^*^^*^^*^	0.46 (0.213)	−7.8
DMN VAN	0.002881	0.1124644 ^*^^*^^*^	0.2428^*^	0.4144332^*^^*^^*^	0.45 (0.20)	6.1
VAN	−0.00047	0.008067524	0.241	−0.01692201	0.46 (0.212)	0
DAN	0.01011	0.08372501^*^	0.2448^*^^*^	0.4122174^*^^*^^*^	0.46 (0.213)	7.9
VAN DAN	0.00508	0.08392439^*^	0.2432^*^	0.412217^*^^*^^*^	0.46 (0.211)	−8.2
	*Fluid cognition*					
	**Repeated k-folds** **Only fMRI rs-FC**		**Repeated k- folds** All covariates		**Lasso at lambda 1 s**	
	**R** ^ **2** ^	**rho**	**R** ^ **2** ^	**rho**	**rho (R** ^**2**^)	**β**
DMN	0.001113	0.01766191	*0.1435*	0.3210988^*^^*^^*^	0.44 (0.20)	0
DMN DAN	0.006383	0.06410616	*0.1447*	0.3257818 ^*^^*^^*^	0.45 (0.20)	−7.5
DMN VAN	0.001623	0.01235055	0.1437	0.3216738^*^^*^^*^	0.31 (0.11)	1.6
VAN	−0.0005418	0.008067524	*0.1439*	0.4010969^*^^*^^*^	0.31 (0.11)	0
DAN	0.009127	0.08372501^*^	0.1459^*^	0.3733809 ^*^^*^^*^	0.32 (0.12)	8.1
VAN DAN	0.003006	0.0416428	*0.1435*	0.3239187^*^	0.31 (0.11)	0

The predictors were within-network connectivity of the DMN, VAN, and DAN and between-network connectivity between these networks. Repeated k-folds cross-validation was performed with 10 folds and 5 repetitions in random samples, with 75% of the data in the training sample (N = 1813) and 25% of the whole sample in the test data (N = 894). See details of these samples in Table S1. Age, sex, MRI manufacturer, income, education, average framewise head displacement (FD), and site were used as covariates for the models that includes all the covariates. Predictive accuracy was also reported for the simplest models that have as an only predictor network connectivity and predicted variable scores in crystallized and fluid abilities. As a measure of prediction accuracy, the table reports the R^2^ for the association between predicted and observed cognitive scores for every predictive model and Spearman's correlation coefficients (rho). LASSO regression was performed as a confirmatory analysis. The results correspond to the models after hyperparameters tunning using kfolds, the table report the Lasso coefficients obtained in the best model at λ.1se and α = 1. The table report the Spearman’s rho for the correlation between predicted and observed cognitive scores of the LASSO regression models, and between brackets the coefficient of determination (R^2^) for the same models. The symbol * denotes the significance levels of the p-values for all the metrics of predictive accuracy (^*^p < 0.05, ^**^p < 0.01, ^***^p < 0.001).

### Cross-validation of the linear models

**Fig. 3:**
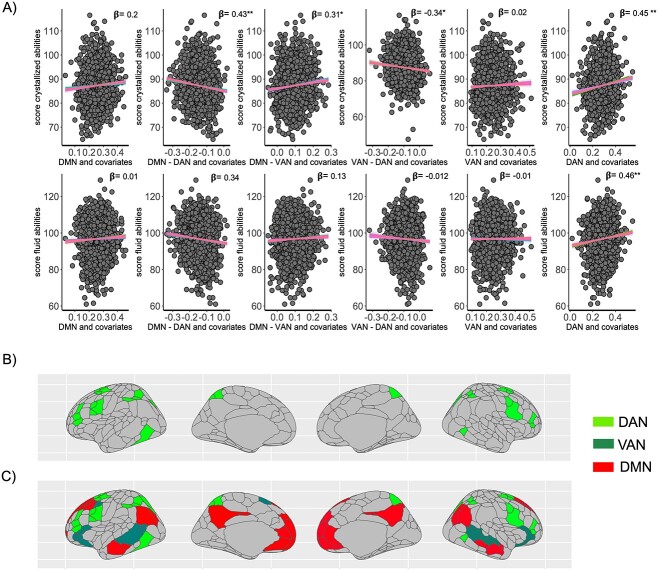
Different functional coupling patterns of the DMN and attentional networks predict child crystalized and fluid abilities in childhood. The figures show in panel A) the scatter plots and superimposed regression lines for the different folds of cross-validated linear regression models (10 folds—5 repetitions) having as predictors within and between-network connectivity of the DAN, VAN, and DMN, and covariates. The shaded area shows the superimposed error bars for the linear regressions of the models having as a predictor within network connectivity of the default mode network (DMN) and between network connectivity between the DMN and dorsal (DAN) and ventral attention networks (VAN) and as predicted outcomes the composite score on crystalized (top) and fluid abilities bottom scatter plots of panel A. Within-network connectivity of the DAN predicts positive change in crystallized and fluid abilities; the same pattern is observed for the models that have as a predictor connectivity between DMN and DAN. In addition, the augmented anticorrelation between the DMN and DAN and VAN DAN is associated with higher scores on crystallized abilities. On the other hand, increased within-network connectivity of the DAN predicts higher scores in crystilized and fluid abilities. The linear multivariate models have age sex MRI scan type, average framewise displacement, income, parent’s education, and site as covariates. P-values were corrected with FDR. Panels B) and C) shows the anatomical projection of the networks predicting fluid (panel B) and crystallized abilities (panel C) based on the predictive models on panel A. The resting-state networks were projected to a cortical surface based on Gordon atlas with 422 parcells.

**Fig. 4:**
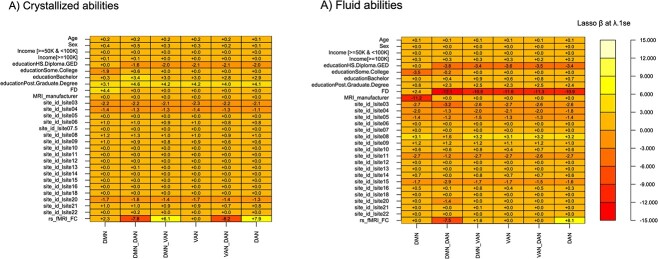
Parental education is an important feature for predicting child’s fluid and crystallized abilities. The figure show the sparse matrix of the cross-validated LASSO regression models having as a outcome the composite scores on crystalized and fluid abilities in A) and B). Variable importance was quantified as the absolute value of the β coefficients after the shrinkage performed by LASSO regularization on the β coefficients. The LASSO model selected was the one that minimizes the error by tuning the hyperparameter at λ.1se. Note in the models that have as outcomes crystallized skills higher β coefficients are the ones corresponding to the predictors assessing brain functional connectivity (FC within or between-network connectivity at DMN, DAN, and VAN). The β coefficients obtained by k-folds coincide with those identified by strong penalization of LASSO regression, compare the β coefficients obtained by cross-validation of the linear models with the two methods kfolds and LASSO in Table 1. Importantly, for crystallized abilities, parental education appears as one of the more important factors in predicting intelligence but also as the third feature for fluid intelligence. Panels A and B show that a higher parental education level (post-graduate degree) is associated with positive differences in crystallized and fluid intelligence (the positive sign of β coefficients for post-graduate level of parental education in A and B). Conversely, lower education level is associated with negative changes in crystallized and fluid abilities; see the negative sign for the β coefficients for the lower levels of parental education (having an HS or high-school diploma).

Next, we randomly split the data into training and testing samples, N = 1813 for training and N = 894 for testing, see features of these samples in Table S3. We trained the regression models and performed cross-validation with k-folds using “glmnet “repeatedcv“ with 10 folds and 5 repetitions by searching the best parameter derived through hyperparameter tuning within a grid using the “tuneGrid” function (cross-validated regression models in panel A of Fig. 3). Cross-validated LASSO regression was performed as a confirmatory analysis. LASSO was cross-validated with 10 folds using “cv.glmnet” searching in a grid of λ that minimized the error at alpha = 1 (see β coefficients in Table 1 and sparse matrix of β coefficients for all linear models n Fig. 4). The best tune model was the one with λ.1se (Table 1 and Fig. 4). We then quantified the prediction accuracy for all the models in the test data (see R-square for the correlation between observed values in the test data and predicted values obtained from training data in Table 1). Given that multicentric studies are prompt to exhibit batch effects, due to different MRI scans and the number of participants at each site in the final sample, we tested the robustness of the results by running additional analyses in random half-split of the final cohort, replication and discovery data N = 1544 and N = 1545, and quantified the bootstrap distribution of the β coefficients for the linear regression models in both random samples (see features of these samples in Table S4). Participants were matched by demographic variables over the 21 sites. For an accurate estimation of the β coefficients of the regression models, we computed the bootstrap sampling distribution for each β coefficient of the linear models with the R package ‘lmboot’. Robust estimation of β coefficients was calculated based on wild bootstrap distributions with 60,000 repetitions. 97.5^th^% and 2.5^th^% of the bootstrap distribution were computed in both random half-splits (Figs. S2 and S3). To assess if the results relate to the presence of nuisance variables in the model or collinearity between multiple factors added to the model, we calculated the models with and without covariates; the estimation of the coefficients has not changed by changing the covariates (compare the results in Figs. S2 and S3 with the resutls reported in Table 1).

### Comparison of β coefficients between linear regression models for crystalized and fluid abilities

To compare the different trends in the predictors for the models having as outcome crystalized and fluid abilities. We compared the β coefficients between multivariate linear regression models by estimating with the bootstrap distributions from 10000 repetitions of standardized β coefficients for the models having as outcomes the average scores in fluid and crystallized abilities in the whole population (N = 2707). The bootstrap distribution of β coefficients for the functional connectivity (FC) factors was standardized based on the formula βs = βr (*σx/σy*) where βs is the standardized coefficient, βr the raw beta coefficient, σx is the standard deviations of the predictor (network FC), and σy the standard deviation of the intelligence scores. The mean values were compared with a one-tilled Wilcoxon test (See Fig. S4). The distances between distributions of standardized β coefficients for every model having as an outcomes crystilized and fluid abilities were compared with two-sample Kolmorogov—Smirnov test in R. As a final step, we tested whether the results obtained remain in the presence of other resting-state networks in the model that had been pointed as important for the emergence of intelligence in adults, as is the case of the frontoparietal network (FPN). For this, LASSO regression models were quantified for all four cognitive outcomes (raw crystallized intelligence score from NIH TBS, crystallized intelligence with picture vocabulary test o composite score in crystallized abilities, composite score on fluid abilities and raw fluid intelligence score from NIH TBS, and average fluid intelligence with shortlist working memory or composite score in fluid abilities) for every model, allFC measures were included together in the same model (see Table S5). Cross-validation was performed using 10 folds using “cv.glmnet” searching in a grid of λ that minimized the error at alpha = 1. In order to test the reliability of the brain-behavior associations showed in these last LASSO regression models (given the higher risk of overfitting because of the increased number of predictors), we computed partial correlation coefficients between every cognitive score and functional connectivity measures for all the target rs-networks corrected by age and FD (head movement). The Spearman partial correlation rho coefficients (ρ), t-statistics, and p-values corrected for multiple comparisons with conservative Bonferroni and reported in Table S6. Partial correlations were computed with the toolbox “ppcor” from R.

### Functional anatomy

The functional anatomy of brain networks involved in fluid and crystalized cognition was plotted using the “ggseg” package implemented in R (Mowinckel and Vidal-Piñeiro 2020), accessible via GitHub: https://github.com/ggseg/ggseg, which builds on “ggplot2.”

## Results

### The anti-correlation between the DAN and DMN and the correlation between DMN and VAN characterize the functional coupling of the DMN and attentional networks in children

We first assessed whether crystallized and fluid abilities would be mirrored by different functional segregation/integration patterns of functional connectivity by looking at within and between network connectivity of the networks of interest. To evaluate whether each network is correlated or anticorrelated, we compared the correlation differences between the within-network connectivity of every network as a seed with the between-network connectivity of the seed network with the other two networks of interest. If the seed within-network connectivity of one network is synchronized with the other two, the differences in Pearson correlation r between the seed network and between-network connectivity of the other networks should be near zero. Conversely, if the seed network expresses an asymmetric functional coupling, the difference in correlation should be near 1 (Fig. 2).

We found that the correlation between within-network connectivity of the VAN and between-network connectivity of the DMN VAN is (r = 0.51) while the correlation within-network connectivity of VAN with DAN VAN between-network connectivity is (r = −0.53); Fig. 2 upper panel C. The difference between Pearson’s *r* calculated by 4,000 bootstrapped repetitions was close to one (x̅ = 1.04, CI 0.99—1.09, *P* < 0. 01). This result shows that increases in VAN within-network connectivity are associated with increased anti-correlation DAN VAN (histogram in Fig. 2 panel C). In addition, the correlation of within-network connectivity of the DMN and between network connectivity of DMN VAN is (r = 0.43), and DMN DAN, between network connectivity is (r = −0.67). The difference between Pearson correlation coefficients r was close to one (x̅ = 1.10, CI 1.06—1.14, *P* < 0. 01) panel A. Note that when the VAN or DMN are the seed networks the differences in Pearson’s correlations *r* is near 1, showing that the sign of the functional coupling of the seed network with between-network connectivity of the other two networks is asymmetric. Notably, the DAN is anti-correlated with DAN VAN and DAN DMN between-connectivity. Whitin network connectivity of the DAN and between-network connectivity of the DMN DAN (r = −0.68), and between VAN DAN (r = −0.51) expressed a correlation difference of (x̅ = −0.16, CI -0.19—0.13, *P* < 0. 01), which is near to zero, and thus denotes a symmetric coupling of the seed network DAN with the DAN DMN and DAN VAN (histogram in Fig. 2 panel B). If we compare the three distributions (lower histograms in Fig. 2), it appears evident that when the seed network is DAN, the differences are more than twenty folds smaller compared with the case in which the seed networks are the DMN in panel A or when the seed network is VAN in panel C. These results show that the DAN is symmetrically anticorrelated with the DMN and VAN. This pattern contrasts with the asymmetric functional coupling of DMN with the VAN. These results show that the functional segregation of the DAN is the main feature of the functional interaction between the DMN, DAN, and VAN in childhood.

### Large-scale communication between the DMN and attentional networks in children distinguishes the neural substrates of crystallized from fluid abilities

Next, we aimed to understand whether intrinsic large-scale coupling measured as within and between FC at the DMN, DAN, and VAN predicts crystallized and fluid abilities. For this, we regressed within and between network connectivity of the DMN, DAN, and VAN in separated models having as predictors the composite scores on crystallized or fluid abilities. We added as covariates age, sex, income, site of the multicentric study, MRI device, and head framewise displacement (FD). We found that crystalized abilities is predicted by between-network connectivity between the DMN, the DAN, and VAN (see Fig. 3). But furthermore, within-network connectivity of the DAN predicts crystallized and fluid abilities. The correlations between functional connectivity and cognition remain significant even in the simplest models in which included as a single factor within or between-network connectivity at the DMN VAN DAN in separated models, having as an outcome either crystallized or fluid abilities in Table 1.

Figure 3 shows the regression lines for the cross-validated linear models in the training data (see Table 1 for prediction accuracy for all the linear models assessed). The results reported in Table 1 are consistent with those obtained when we estimated the β coefficients by 60.0000 bootstrap replications in discovery and replication datasets (see Figs. S2, S3, and Table S2). Furthermore, the results obtained in both half-split samples were consistent with those obtained by cross-validation of the linear regression models with k-folds and LASSO. In summary, the results above show that large-scale coupling between DMN and attentional networks distinguish brain-behavior associations having crystallized abilities as an outcome. On the other hand, fluid abilities express a trend than crystallized abilities to be predicted by within-network connectivity of the DAN. To compare the different trends in brain-behavior associations between models with different outcomes see in Fig. S4 the boostrap distribution distances of the standardized β coefficients for the factors measuring functional connectivity in the regression models having as predicted variables crystallized vs fluid abilities.

To estimate the importance of the factors measuring functional connectivity relative to the weight of socioeconomic predictors on crystallized and fluid abilities, we analyzed the sparse matrix of β coefficients from the cross-validated LASSO regression models (Fig. 4). The functional connectivity factors (either within or between network connectivity at DAN VAN DMN) in all the LASSO models having as an outcomes crystallized and fluid abilities were more important as a variable than environmental variables such as parental education, which indeed, appears as the second feature of the model predicting crystallized abilities (Fig. 4A). Note that for the models having as an outcome fluid ability, having a parent with a post-graduate degree correlates with higher cognitive performance (Fig. 4B), while the lower grades (having a high-school diploma of lower) correlate with negative trends in the brain-behaviour associations involving both crystallized and fluid abilities.

Because the frontoparietal network (FPN) has been pointed as an important network to the emergence of fluid abilities in adults (Finn et al. 2015), we finally searched to understand whether when we include all the FC predictors together in the same predictive model (DMN DAN VAN and FPN) with socioeconomic factors we have similar results than the above described (in Fig. 3). Even though some of the β coefficients assessing the association between FPN connectivity and intelligence do not shrink to zero in the cross-validated LASSO regression models (Table S5), we found that the partial correlations between FPN and intelligence did not reached statistical significance after Bonferroni multiple comparison correction (Table S6). On the other hand, the association between DMM and attentional networks remains significant in both, the LASSO regression models including all the FC factors together in the same model, and partial correlation analysis (see Tables S5 and S6).

## Discussion

We show that within-network connectivity of the DAN and large-scale coupling between DMN and attentional networks predict crystallized abilities. In contrast, fluid abilities are predicted by functional connectivity of a more focal functional network configuration centered at the frontoparietal nodes of the dorsal attention network. We show that crystallized and fluid abilities are functionally anchored at nodes of the frontoparietal dorsal attention network, but expressing different trends in the brain-behavior associations depending of whether only DAN predict cognition (in fluid abilities), or it is also predicted by the functional connectivity of the attentional networks with the DMN, as is the case of crystilized abilities. Our results challenge the current view of a dissociated organization of crystallized and fluid abilities, suggesting that while some neural substrates are dissociated, the frontoparietal DAN nodes are central to the emergence of fluid and crystallized abilities in children. These results may suggest that the frontoparietal nodes of the DAN could be a future target of functional enhance of learning for experience-based and analytical reasoning abilities in children.

Previous evidence supports the idea that the anti-correlated intrinsic organization of resting-state networks is needed for effective behavior and cognition. On the other hand, previous studies have shown that during child development, the human connectome resembles more a mosaic of correlated and anticorrelated brain regions than a homogeneous pattern of functionally segregated brain networks as is observed in the adult connectome (Chai et al. 2014). In agreement with the literature, we found that the DMN is anti-correlated with the DAN (see Fig. 2), concordant with (Keller et al. 2013; Andrews-Hanna et al. 2014), and that the VAN and DAN are also anti-correlated (Fox et al. 2005). We interpret our findings as evidence that this functional organization renders an effective functional organization for long-range information routing that may facilitate to the dorsal frontoparietal network manage the computational resources to control the emergence of abstract reasoning (Santarnecchi et al. 2017). On the other hand, we found that the VAN is correlated with DMN. This is in line with evidence showing that correlations between DMN and task-positive networks reverse from positive in children to an anticorrelated configuration in adults (Chai et al. 2014). Therefore we interpret these results as a step of maturation of the attentional system that migrates towards a more segregated functional organization between task-positive and task-negative networks, as in the adult functional organization (Kelly et al. 2008). In line with this interpretation, recent evidence has shown that DMN is not anticorrelated but correlated with VAN in childhood, and importantly, the strength of network connectivity between DMN and VAN is functionally relevant because has been shown is modulated by environmental factors such as positive parenting (Rakesh et al. 2021). Our results expand these findings showing that DMN and attentional networks express an asymmetric coupling in children, possibly due to different a function of the DAN and VAN in the system-level brain integration relevant for preparing the brain for the adult configuration, and congruent with an effective emergence of complex cognitive processes in the lifespan. But furthermore, besides the pattern of anticorrelated networks, we found that FPN does not appeared as an important feature for the emergence of intelligence in the childhood (see Tables S5 and S6), this contrast with the relevance of the FPN explaining intelligence in adults (Finn et al. 2015). This evidence can be interpreted in the context of the still immature stage of development of the FPN in the preadolescence (Blakemore and Choudhury 2006), in this context, the functional relevance of DMN and attentional networks in children might compensate the underdevelopment of the FPN supporting higher cognitive functions in children (Chen et al. 2023). In this context, the prediction of the anticorrelation between DMN and attentional networks on intelligence of our study, can be interpreted as the use of alternative networks to accomplish similar functions than during adulthood in the context of the still immature development of the FPN in preadolescents. Remains to be established this possible functional asymmetry between the DMN and attentional networks and the development of FPN might be modulated by contextual or environmental factors during critical periods of neurodevelopment, as preadolescence is.

We found that increased correlation between the DMN and VAN, anti-correlation between DMN and DAN and between the VAN and DAN predicts crystallized abilities. Moreover, within-network connectivity of the DAN predicted both crystallized and fluid abilities. The evidence that DAN within-network connectivity correlates with both, crystallized and fluid intelligence (Fig. 3) is in line with previous evidence supporting that DAN connectivity is associated with attention problems in children (Rohr et al. 2017). But furthermore, these results are in accord with the evidence supporting that maintaining attention span is important basin cognitive function supporting problem-solving skills (Schweizer et al. 2005). We thus expand these previous findings showing that connectivity at the DAN plays a central role predicting performance in the complex mental processes needed for the emergence of fluid and crystallized cognition. Our results suggest that crystallized skills in children depend on the recovery of mental representations that emerge from the functional interaction of task-positive networks as DAN and VAN with task-negative networks as the DMN. This idea agrees with the recent view of the role of the DMN as a functionally related network to high-order cognitive processes (Smallwood et al. 2021), facilitating the reactivation of complex abstract mental processes needed for attention engagement during reading comprehension (Song et al. 2021). In this line, increased segregation of the DMN in children has been linked to a top-down cognitive control (Pines et al. 2022). Future works might be focus on understanding the clinical and neurodevelopmental trajectories of the functional interactions between task-negative and task-positive networks in comparation with adults, and during diseases.

Evidence shown that inconsistent with a purely task-negative view of DMN function, the DMN expresses strong associations with attentional networks during an attentional task (Williams et al. 2022), and also in a more complex cognitive task of decision-making when decisions depend on prior experience (Sormaz et al. 2018; Turnbull et al. 2019). Our results show that more than the DMN itself, is the functional coupling of the DMN and attentional networks what is relevant for higher cognitive functions in children, notably for the emergence of crystallized abilities. This evidence aligns with previous works in the same population showing that the anti-correlation between DMN and DAN is associated with attentional problems in children (Owens et al. 2020). In line with this, we show in Fig. 3 that the anti-correlation between attentional networks relates to crystallized abilities; these results are in line with seminal study on the functional organization of the attentional networks described in the resting-state (Fox et al. 2006), which with the same functional organization turns to be a positive correlation during a task (Williams et al. 2022). Finally, we expand these findings showing that the triple functional interaction between DMN, DAN, and the VAN is not only related to basic cognitive functions such as vigilance and attention but also closely tied to the emergence of complex mental processes in children such as abstract reasoning.

We showed that the within-network connectivity of the DAN predicts fluid abilities (see Figs. 3, S2 and S3). Recent evidence using graph lesion-deficit mapping analysis showed that fluid intelligence relies on a prefrontal network (Cipolotti et al. 2023). On the other hand, the DAN in adults involves functional activation of inferior parietal brain regions, which is in line with evidence supporting that intelligence relies on the large-scale integration of a frontoparietal network (Colom et al. 2010). To explain the discrepancy of the DAN nodes identified in our study, we also quantified fluid abilities as a composite score involving shorting list working memory. The inferior parietal nodes of the DAN associated with fluid intelligence scores in our study may be related to the working memory-related brain activation (Colom et al. 2010). According to this idea, evidence shows that the progressive engagement of the prefrontal cortex and inferior parietal lobule increases with reasoning abilities (Wendelken et al. 2015). Our evidence supports the idea that fluid abilities relay to the function of DAN frontoparietal nodes, and gives insight into training attention during childhood may improve abstract and analytical skills.

We found that parental education is also a relevant feature in explaining crystallized and fluid abilities in children (Fig. 4). Previous evidence has shown that positive parenting is more important in explaining crystallized than fluid intelligence in children (Alves et al. 2017; Rindermann and Ceci 2018). Our results shows that parental education is a second-order importance factor predicting crystallized abilities in children compared with brain functional connectivity factors. We must acknowledge that education attainment in our sample, is higher than the educational attainment of the US population (Ryan and Bauman 2016). However, our measure of education attainment coincides with the one reported in the whole population of the ABCD study (Heeringa and Berglund 2020), and therefore it is unlikely that we introduce bias by sampling the current study population. Future studies may focus on the possible interaction between parental education and intrinsic connectivity of the DMN and attentional networks to attempt understand whether education shape brain functional connections to predict children’s cognition and different neurodevelopment trajectories.

Finally, our results show that crystallized and fluid abilities have commonalities in their neural substrate. Based on these similarities, and recent behavioral evidence in adults supporting their common trends in decay across the lifespan (Tucker-Drob et al. 2022), it seems unlikely that crystallized abilities would compensate for fluid intelligence loss with aging. Instead, the fact that maintenance of crystallized intelligence in the elderly and spared in Alzheimer’s disease would be explained as a cause of their neural substrates, with a more distributed functional organization for the network supporting crystallized abilities, and consequently, more resilient to pathology than for fluid abilities. With caution, our study might suggest that training functional interactions between attentional networks and DMN may be a target to improve learning during critical periods of neurodevelopment, as during pre-adolescence, to prevent cognitive decline in the lifespan.

## Supplementary Material

Supplementary_material_updated_tgad015

## Data Availability

Data used in the preparation of this article were obtained from the Adolescent Brain Cognitive Development^SM ^ (ABCD) Study (https://abcdstudy.org), held in the NIMH Data Archive (NDA). This is a multisite, longitudinal study designed to recruit more than 10,000 children age 9–10 and follow them over 10 years into early adulthood. The ABCD Study® is supported by the National Institutes of Health and additional federal partners under award numbers U01DA041048, U01DA050989, U01DA051016, U01DA041022, U01DA051018, U01DA051037, U01DA050987, U01DA041174, U01DA041106, U01DA041117, U01DA041028, U01DA041134, U01DA050988, U01DA051039, U01DA041156, U01DA041025, U01DA041120, U01DA051038, U01DA041148, U01DA041093, U01DA041089, U24DA041123, U24DA041147. A full list of supporters is available at https://abcdstudy.org/federal-partners.html. A listing of participating sites and a complete listing of the study investigators can be found at https://abcdstudy.org/consortium_members/. ABCD consortium investigators designed and implemented the study and/or provided data but did not necessarily participate in the analysis or writing of this report. This manuscript reflects the views of the authors and may not reflect the opinions or views of the NIH or ABCD consortium investigators. The ABCD data repository grows and changes over time. The ABCD data used in this report is specified at http://dx.doi.org/10.15154/bgph-eg22.

## References

[ref1] Akshoomoff N, Beaumont JL, Bauer PJ, Dikmen SS, Gershon RC, Mungas D, Slotkin J, Tulsky D, Weintraub S, Zelazo PD, et al. VIII. NIH toolbox cognition battery (CB): composite scores of crystallized, fluid, and overall cognition. Monogr Soc Res Child Dev. 2013:78(4):119–132. 23952206 10.1111/mono.12038 PMC4103789

[ref2] Alves AF, Gomes CMA, Martins A, Almeida LS. Cognitive performance and academic achievement: how do family and school converge? Eur J Educ Psychol. 2017:10(2):49–56.

[ref3] Andrews-Hanna JR, Smallwood J, Spreng RN. The default network and self-generated thought: component processes, dynamic control, and clinical relevance. Ann N Y Acad Sci. 2014:1316(1):29–52. 24502540 10.1111/nyas.12360 PMC4039623

[ref4] Babyak MA . What you see may not be what you get: a brief, nontechnical introduction to overfitting in regression-type models. Psychosom Med. 2004:66(3):411–421. 15184705 10.1097/01.psy.0000127692.23278.a9

[ref5] Blair C . How similar are fluid cognition and general intelligence? A developmental neuroscience perspective on fluid cognition as an aspect of human cognitive ability. Behav Brain Sci. 2006:29(2):109–125 discussion 125-160. 16606477 10.1017/S0140525X06009034

[ref6] Blakemore S-J, Choudhury S. Development of the adolescent brain: implications for executive function and social cognition. J Child Psychol Psychiatry. 2006:47(3-4):296–312. 16492261 10.1111/j.1469-7610.2006.01611.x

[ref7] Casey BJ, Cannonier T, Conley MI, Cohen AO, Barch DM, Heitzeg MM, Soules ME, Teslovich T, Dellarco DV, Garavan H, et al. The Adolescent Brain Cognitive Development (ABCD) study: imaging acquisition across 21 sites. Dev Cogn Neurosci. 2018:32:43–54. 29567376 10.1016/j.dcn.2018.03.001 PMC5999559

[ref8] Cattell RB . Theory of fluid and crystallized intelligence: a critical experiment. J Educ Psychol. 1963:54(1):1–22. 10.1037/h0024654 6043849

[ref9] Chai XJ, Ofen N, Gabrieli JD, Whitfield-Gabrieli S. Selective development of anticorrelated networks in the intrinsic functional organization of the human brain. J Cogn Neurosci. 2014:26(3):501–513. 24188367 10.1162/jocn_a_00517 PMC4175987

[ref10] Chen M, He Y, Hao L, Xu J, Tian T, Peng S, Zhao G, Lu J, Zhao Y, Zhao H, et al. Default mode network scaffolds immature frontoparietal network in cognitive development. Cereb Cortex. 2023:33(9):5251–5263. 36320154 10.1093/cercor/bhac414 PMC10152054

[ref11] Cipolotti L, Ruffle JK, Mole J, Xu T, Hyare H, Shallice T, Chan E, Nachev P. Graph lesion-deficit mapping of fluid intelligence. Brain. 2023:146(1):167–181. 36574957 10.1093/brain/awac304 PMC9825598

[ref12] Colom R, Karama S, Jung RE, Haier RJ. Human intelligence and brain networks. Dialogues Clin Neurosci. 2010:12(4):489–501. 21319494 10.31887/DCNS.2010.12.4/rcolom PMC3181994

[ref13] Corbetta M, Patel G, Shulman GL. The reorienting system of the human brain: from environment to theory of mind. Neuron. 2008:58(3):306–324. 18466742 10.1016/j.neuron.2008.04.017 PMC2441869

[ref14] Cosgrove KT, McDermott TJ, White EJ, Mosconi MW, Thompson WK, Paulus MP, Cardenas-Iniguez C, Aupperle RL. Limits to the generalizability of resting-state functional magnetic resonance imaging studies of youth: an examination of ABCD Study® baseline data. Brain Imaging Behav. 2022:16(4):1919–1925. 35552993 10.1007/s11682-022-00665-2 PMC9296258

[ref15] Deary IJ, Penke L, Johnson W. The neuroscience of human intelligence differences. Nat Rev Neurosci. 2010:11(3):201–211. 20145623 10.1038/nrn2793

[ref16] Deco G, Tononi G, Boly M, Kringelbach ML. Rethinking segregation and integration: contributions of whole-brain modelling. Nat Rev Neurosci. 2015:16(7):430–439. 10.1038/nrn3963 26081790

[ref17] Dhamala E, Jamison KW, Jaywant A, Dennis S, Kuceyeski A. Distinct functional and structural connections predict crystallised and fluid cognition in healthy adults. Hum Brain Mapp. 2021:42(10):3102–3118. 33830577 10.1002/hbm.25420 PMC8193532

[ref18] Dixon ML, Andrews-Hanna JR, Spreng RN, Irving ZC, Mills C, Girn M, Christoff K. Interactions between the default network and dorsal attention network vary across default subsystems, time, and cognitive states. NeuroImage. 2017:147:632–649. 28040543 10.1016/j.neuroimage.2016.12.073

[ref19] Finn E, Shen X, Scheinost D, Rosenberg MD, Huang J, Chun MM, Papademetris X, Constable RT. Functional connectome fingerprinting: identifying individuals using patterns of brain connectivity. Nat Neurosci. 2015:18(11):1664–1671. 10.1038/nn.4135. 26457551 PMC5008686

[ref20] Fox MD, Snyder AZ, Vincent JL, Corbetta M, Van Essen DC, Raichle ME. The human brain is intrinsically organized into dynamic, anticorrelated functional networks. Proc Natl Acad Sci. 2005:102(27):9673–9678. 15976020 10.1073/pnas.0504136102 PMC1157105

[ref21] Fox MD, Corbetta M, Snyder AZ, Vincent JL, Raichle ME. Spontaneous neuronal activity distinguishes human dorsal and ventral attention systems. Proc Natl Acad Sci. 2006:103(26):10046–10051. 16788060 10.1073/pnas.0604187103 PMC1480402

[ref22] Fransson P, Strindberg M. Brain network integration, segregation and quasi-periodic activation and deactivation during tasks and rest. NeuroImage. 2023:268:119890. 36681135 10.1016/j.neuroimage.2023.119890

[ref23] Garavan H, Bartsch H, Conway K, Decastro A, Goldstein R, Heeringa S, Jernigan T, Potter A, Thompson W, Zahs D. Recruiting the ABCD sample: design considerations and procedures. Dev Cogn Neurosci. 2018:32:16–22. 29703560 10.1016/j.dcn.2018.04.004 PMC6314286

[ref24] Geake JG, Hansen PC. Functional neural correlates of fluid and crystallized analogizing. NeuroImage. 2010:49(4):3489–3497. 19761849 10.1016/j.neuroimage.2009.09.008

[ref25] Gordon EM, Laumann TO, Adeyemo B, Huckins JF, Kelley WM, Petersen SE. Generation and evaluation of a cortical area parcellation from resting-state correlations. Cereb Cortex. 2016:26(1):288–303. 25316338 10.1093/cercor/bhu239 PMC4677978

[ref26] Hackman DA, Farah MJ. Socioeconomic status and the developing brain. Trends Cogn Sci. 2009:13(2):65–73. 19135405 10.1016/j.tics.2008.11.003 PMC3575682

[ref27] Hagler DJ Jr, Hatton S, Cornejo MD, Makowski C, Fair DA, Dick AS, Sutherland MT, Casey B, Barch DM, Harms MP, et al. Image processing and analysis methods for the Adolescent Brain Cognitive Development study. NeuroImage. 2019:202:116091. 31415884 10.1016/j.neuroimage.2019.116091 PMC6981278

[ref28] Heaton RK, Akshoomoff N, Tulsky D, Mungas D, Weintraub S, Dikmen S, Beaumont J, Casaletto KB, Conway K, Slotkin J, et al. Reliability and validity of composite scores from the NIH toolbox cognition battery in adults. J Int Neuropsychol Soc. 2014:20(6):588–598. 24960398 10.1017/S1355617714000241 PMC4103963

[ref29] Heeringa S, Berglund P. A guide for population-based analysis of the Adolescent Brain Cognitive Development (ABCD) study baseline data. BioRxiv. 2020:2020-02.

[ref30] Horn JL . Organization of abilities and the development of intelligence. In: Eysenck HJ, editors. The measurement of intelligence Dordrecht. Netherlands: Springer; 1973. pp. 137–154

[ref31] Keller CJ, Bickel S, Honey CJ, Groppe DM, Entz L, Craddock RC, Lado FA, Kelly C, Milham M, Mehta AD. Neurophysiological investigation of spontaneous correlated and anticorrelated fluctuations of the BOLD signal. J Neurosci. 2013:33(15):6333–6342. 23575832 10.1523/JNEUROSCI.4837-12.2013 PMC3652257

[ref32] Kelly AC, Uddin LQ, Biswal BB, Castellanos FX, Milham MP. Competition between functional brain networks mediates behavioral variability. NeuroImage. 2008:39(1):527–537. 17919929 10.1016/j.neuroimage.2007.08.008

[ref33] Luciana M, Bjork JM, Nagel BJ, Barch DM, Gonzalez R, Nixon SJ, Banich MT. Adolescent neurocognitive development and impacts of substance use: overview of the Adolescent Brain Cognitive Development (ABCD) baseline neurocognition battery. Dev Cogn Neurosci. 2018:32:67–79. 29525452 10.1016/j.dcn.2018.02.006 PMC6039970

[ref34] Melkumova LE, Shatskikh SY. Comparing ridge and LASSO estimators for data analysis. Procedia Eng. 2017:201:746–755.

[ref35] Menon V . Developmental pathways to functional brain networks: emerging principles. Trends Cogn Sci. 2013:17(12):627–640. 24183779 10.1016/j.tics.2013.09.015

[ref36] Mowinckel AM, Vidal-Piñeiro D. Visualization of brain statistics with R packages ggseg and ggseg3d. Adv Methods Pract Psychol Sci. 2020:3(4):466–483.

[ref37] Nielson DM, Pereira F, Zheng CY, Migineishvili N, Lee JA, Thomas AG, Bandettini PA. Detecting and harmonizing scanner differences in the ABCD study-annual release 1.0. 2018: BioRxiv.309260.

[ref38] Owens MM, Yuan D, Hahn S, Albaugh M, Allgaier N, Chaarani B, Potter A, Garavan H. Investigation of psychiatric and neuropsychological correlates of default mode network and dorsal attention network Anticorrelation in children. Cereb Cortex. 2020:30(12):6083–6096. 32591777 10.1093/cercor/bhaa143 PMC8086768

[ref39] Pines AR, Larsen B, Cui Z, Sydnor VJ, Bertolero MA, Adebimpe A, Alexander-Bloch AF, Davatzikos C, Fair DA, Gur RC, et al. Dissociable multi-scale patterns of development in personalized brain networks. Nat Commun. 2022:13(1):2647. 35551181 10.1038/s41467-022-30244-4 PMC9098559

[ref40] Power JD, Mitra A, Laumann TO, Snyder AZ, Schlaggar BL, Petersen SE. Methods to detect, characterize, and remove motion artifact in resting state fMRI. NeuroImage. 2014:84:320–341. 23994314 10.1016/j.neuroimage.2013.08.048 PMC3849338

[ref41] Rakesh D, Seguin C, Zalesky A, Cropley V, Whittle S. Associations between Neighborhood disadvantage, resting-state functional connectivity, and behavior in the Adolescent Brain Cognitive Development study: the moderating role of positive family and school environments. Biol Psychiatry: Cogn Neurosci Neuroimaging. 2021:6(9):877–886. 33771727 10.1016/j.bpsc.2021.03.008

[ref42] Ramchandran K, Zeien E, Andreasen NC. Distributed neural efficiency: intelligence and age modulate adaptive allocation of resources in the brain. Trends Neurosci. Educ. 2019:15:48–61. 31176471 10.1016/j.tine.2019.02.006

[ref43] Rindermann H, Ceci SJ. Parents’ education is more important than their wealth in shaping their children’s intelligence: results of 19 samples in seven countries at different developmental levels. J Educ Gift. 2018:41(4):298–326.

[ref44] Rohr CS, Vinette SA, Parsons KAL, Cho IYK, Dimond D, Benischek A, Lebel C, Dewey D, Bray S. Functional connectivity of the dorsal attention network predicts selective attention in 4-7 year-old girls. Cereb Cortex. 2017:27(9):4350–4360. 27522072 10.1093/cercor/bhw236

[ref45] Román FJ, Abad FJ, Escorial S, Burgaleta M, Martínez K, Álvarez-Linera J, Quiroga M, Karama S, Haier RJ, Colom R. Reversed hierarchy in the brain for general and specific cognitive abilities: a morphometric analysis. Hum Brain Mapp. 2014:35(8):3805–3818. 24677433 10.1002/hbm.22438 PMC6869303

[ref45a] Ryan CL, Bauman K. Educational Attainment in the United States: 2015. Current Population Reports, Suitland, MD: U.S. Census Bureau; 2016.

[ref46] Santarnecchi E, Emmendorfer A, Tadayon S, Rossi S, Rossi A, Pascual-Leone A. Network connectivity correlates of variability in fluid intelligence performance. Intelligence. 2017:65:35–47.

[ref47] Scheinost D, Noble S, Horien C, Greene AS, Lake EM, Salehi M, Gao S, Shen X, O'Connor D, Barron DS, et al. Ten simple rules for predictive modeling of individual differences in neuroimaging. NeuroImage. 2019:193:35–45. 30831310 10.1016/j.neuroimage.2019.02.057 PMC6521850

[ref48] Schurz M, Maliske L, Kanske P. Cross-network interactions in social cognition: a review of findings on task related brain activation and connectivity. Cortex. 2020:130:142–157. 10.1016/j.cortex.2020.05.006. 32653744

[ref49] Schweizer K, Moosbrugger H, Goldhammer F. The structure of the relationship between attention and intelligence. Intelligence. 2005:33(6):589–611.

[ref50] Scott EP, Sorrell A, Benitez A. Psychometric properties of the NIH toolbox cognition battery in healthy older adults: reliability, validity, and agreement with standard neuropsychological tests. J Int Neuropsychol Soc. 2019:25(08):857–867. 31256769 10.1017/S1355617719000614 PMC6733640

[ref51] Simpson-Kent IL, Fuhrmann D, Bathelt J, Achterberg J, Borgeest GS, Kievit RA, CALM Team. Neurocognitive reorganization between crystallized intelligence, fluid intelligence and white matter microstructure in two age-heterogeneous developmental cohorts. Dev Cogn Neurosci. 2020:41:100743. 31999564 10.1016/j.dcn.2019.100743 PMC6983934

[ref52] Smallwood J, Bernhardt BC, Leech R, Bzdok D, Jefferies E, Margulies DS. The default mode network in cognition: a topographical perspective. Nat Rev Neurosci. 2021:22(8):503–513. 34226715 10.1038/s41583-021-00474-4

[ref53] Song H, Finn ES, Rosenberg MD. Neural signatures of attentional engagement during narratives and its consequences for event memory. Proc Natl Acad Sci. 2021:118(33):e2021905118. 34385312 10.1073/pnas.2021905118 PMC8379980

[ref54] Sormaz M, Murphy C, Wang H-t, Hymers M, Karapanagiotidis T, Poerio G, Margulies DS, Jefferies E, Smallwood J. Default mode network can support the level of detail in experience during active task states. Proc Natl Acad Sci. 2018:115(37):9318–9323. 30150393 10.1073/pnas.1721259115 PMC6140531

[ref55] Spreng RN, Stevens WD, Viviano JD, Schacter DL. Attenuated anticorrelation between the default and dorsal attention networks with aging: evidence from task and rest. Neurobiol Aging. 2016:45:149–160. 27459935 10.1016/j.neurobiolaging.2016.05.020 PMC5003045

[ref55a] Tomasi D, Volkow ND. Associations of family income with cognition and brain structure in USA children: prevention implications. Mol Psychiatry. 2021:26(11):6619–6629. 33990770 10.1038/s41380-021-01130-0 PMC8590701

[ref56] Tomasi D, Volkow ND. Measures of brain connectivity and cognition by sex in US children. JAMA Netw Open. 2023:6(2):e230157. 36809470 10.1001/jamanetworkopen.2023.0157 PMC9945095

[ref56a] Tucker-Drob EM, de la Fuente J, Köhncke Y, Brandmaier AM, Nyberg L, Lindenberger U. A strong dependency between changes in *fluid* and crystallized abilities in human cognitive *aging*. Sci Adv. 2022:8(5). 10.1126/sciadv.abj2422 PMC8809681 35108051

[ref57] Turnbull A, Wang H, Murphy C, Ho N, Wang X, Sormaz M, Karapanagiotidis T, Leech R, Bernhardt B, Margulies D, et al. Left dorsolateral prefrontal cortex supports context-dependent prioritisation of off-task thought. Nat Commun. 2019:10(1):3816. 31444333 10.1038/s41467-019-11764-y PMC6707151

[ref58] Varoquaux G, Raamana PR, Engemann DA, Hoyos-Idrobo A, Schwartz Y, Thirion B. Assessing and tuning brain decoders: cross-validation, caveats, and guidelines. NeuroImage. 2017:145(Pt B):166–179. 27989847 10.1016/j.neuroimage.2016.10.038

[ref59] Volkow ND, Koob GF, Croyle RT, Bianchi DW, Gordon JA, Koroshetz WJ, Pérez-Stable EJ, Riley WT, Bloch MH, Conway K, et al. The conception of the ABCD study: from substance use to a broad NIH collaboration. Dev Cogn Neurosci. 2018:32:4–7. 29051027 10.1016/j.dcn.2017.10.002 PMC5893417

[ref60] Wang R, Liu M, Cheng X, Wu Y, Hildebrandt A, Zhou C. Segregation, integration, and balance of large-scale resting brain networks configure different cognitive abilities. Proc Natl Acad Sci. 2021:118(23):e2022288118. 34074762 10.1073/pnas.2022288118 PMC8201916

[ref61] Weintraub S, Dikmen SS, Heaton RK, Tulsky DS, Zelazo PD, Bauer PJ, Carlozzi NE, Slotkin J, Blitz D, Wallner-Allen K, et al. Cognition assessment using the NIH toolbox. Neurology. 2013:80(Issue 11, Supplement 3):S54–S64. 23479546 10.1212/WNL.0b013e3182872ded PMC3662346

[ref62] Wendelken C, Ferrer E, Whitaker KJ, Bunge SA. Fronto-parietal network reconfiguration supports the development of reasoning ability. Cereb Cortex. 2015:26(5):2178–2190. 25824536 10.1093/cercor/bhv050 PMC4830293

[ref63] Williams KA, Numssen O, Hartwigsen G. Task-specific network interactions across key cognitive domains. Cereb Cortex. 2022:32(22):5050–5071. 35158372 10.1093/cercor/bhab531 PMC9667178

