# Supplementary material for: Different patterns of intrinsic functional connectivity at the default mode and attentional networks predict crystalized and fluid abilities in childhood
Source: Cereb Cortex Commun. 2023 Aug 17;4(3):tgad015. doi: 10.1093/texcom/tgad015 (PMC10477707; doi:10.1093/texcom/tgad015)
Supplement: Supplementary_material_updated_tgad015 [file supplementary_material_updated_tgad015.pdf]

Supplementary material

	Overall (N=2707)
**Age**	
Mean (SD)	120 (7.59)
Median [Min, Max]	120 [107, 132]
**Age (months first session)**	
Girls	1405 (51.9%)
Boys	1302 (48.1%)
**Household Income (year) in USD**	
[<50K]	1500 (55.4%)
[>=50K & <100K]	812 (30.0%)
[>=100K]	395 (14.6%)
**Parents_H_Degree**	
< HS Diploma	115 (4.2%)
HS Diploma/GED	249 (9.2%)
Some College	400 (14.8%)
Bachelor	1013 (37.4%)
Post Graduate Degree	930 (34.4%)
**MRI manufacturer**	
GE MEDICAL SYSTEMS	762 (28.1%)
SIEMENS	1945 (71.9%)
**Average framewise displacement in mm**	
Mean (SD)	0.106 (0.0434)
Median [Min, Max]	0.0988 [0.0196, 0.200]
**Fluid intelligence Score NIH**	
Mean (SD)	94.2 (9.68)
Median [Min, Max]	95.0 [59.0, 123]
**Crystilized intelligence Score NIH**	
Mean (SD)	88.1 (6.80)
Median [Min, Max]	88.0 [59.0, 115]
**Picture Vocabulary Test Score NIH**	
Mean (SD)	86.3 (7.98)
Median [Min, Max]	86.0 [36.0, 118]
**NIH List Sorting Working Memory Score**	
Mean (SD)	99.0 (11.0)
Median [Min, Max]	101 [36.0, 136]
**Composite crystilized cognition**	
Mean (SD)	87.2 (7.20)
Median [Min, Max]	86.5 [47.5, 117]
**Composite fluid cognition**	
Mean (SD)	96.6 (9.26)
Median [Min, Max]	97.0 [55.0, 129]

Table S1 *The table reports the main features of the population in the whole cohort of the study N=2707 child's independent of their clinical status*

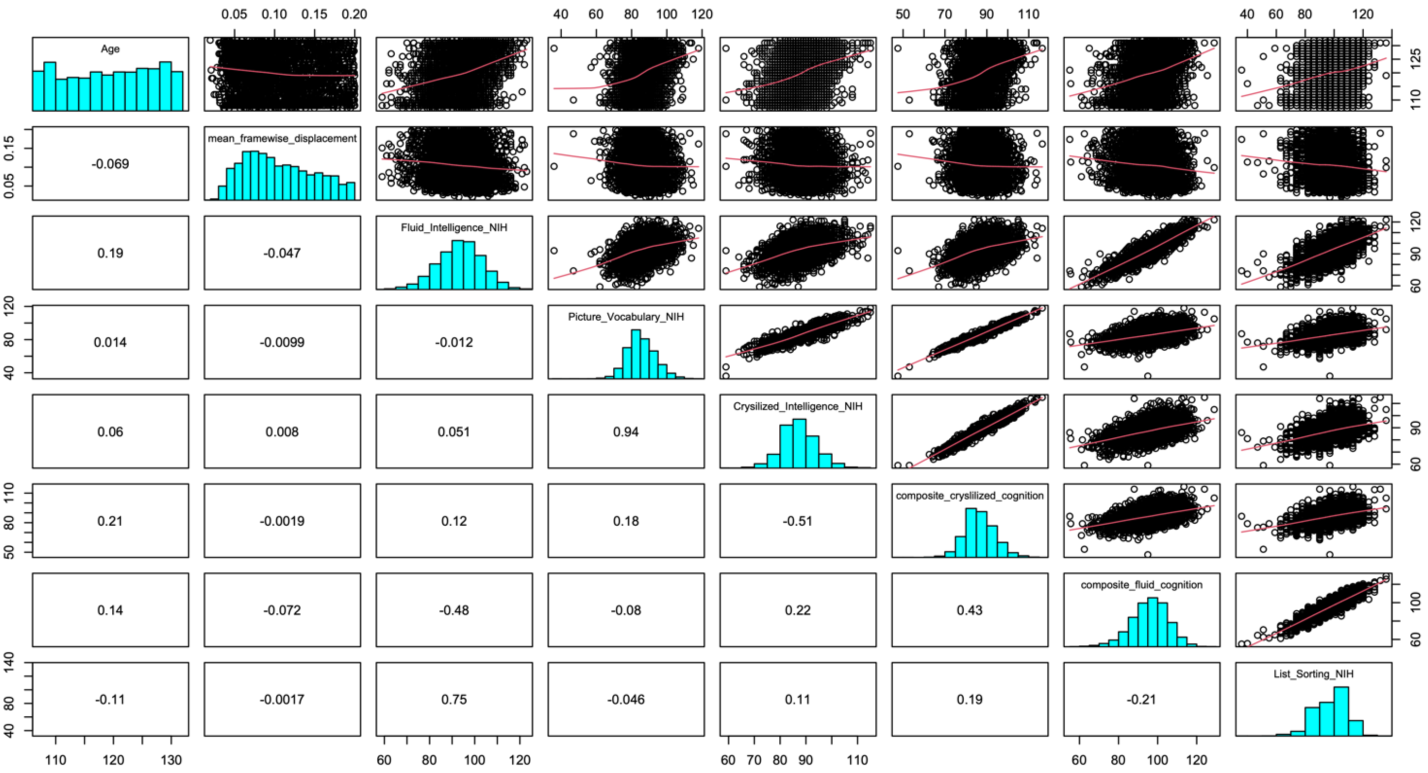

Figure S1. The figure shows the pairwise Spearman correlation coefficients between cognitive scores of interests. Crystalized intelligence NIH raw score strongly correlates with picture vocabulary test ("nihtbx_cryst_uncorrected") while fluid intelligence raw NIH score ("nihtbx_fluidcomp_uncorrected") correlates with List sorting working memory test. These results support that richest vocabulary repertoire is highly correlated with crystallized intelligence while executive functions and working memory is a good proxy of fluid intelligence. Note that individual scores on list sorting NIH score and picture vocabulary test are more correlated in our sample with fluid and crystallized raw scores respectively. This confirm that verbal skills are tightly related to crystallized abilities and executive functions/working memory with fluid abilities. To assess the robustness of the brain-behavior associations for each dimension of intelligence, the predictive models were performed in both, the original raw composite score of crystallized and fluid intelligence from the NIH toolbox and scores obtained by averaging intelligence raw scores of the NIH toolbox with individual cognitive evaluations assessing measures language skills and verbal intellect (picture vocabulary test) and executive functions and working memory (List sorting working memory), see Table S4.

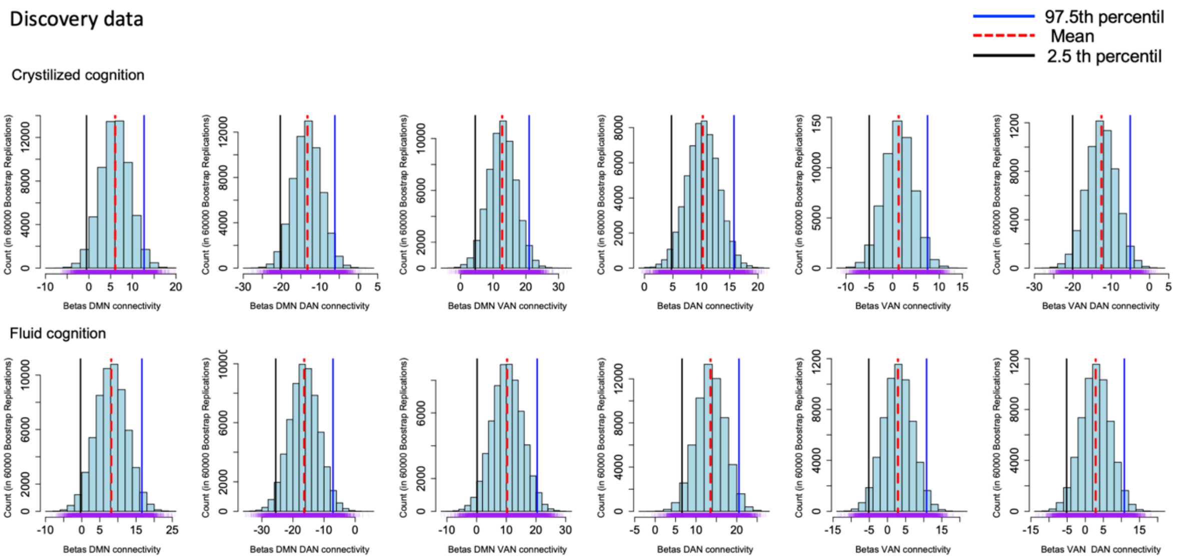

Figure S2 *For internal validation of multivariate regression models the β coefficients were estimated by bootstrapping the sample 60000 times in two random half-splits of the final population (N=1353 discovery, and N=1354 replication)*. The histograms show the *distribution of β coefficients for the linear models in the discovery data set. The confidence intervals for the estimation of the coefficients by 60000 bootstrap repetitions for the models in crystalized and fluid abilities are between the black and blue solid lines (see supplementary table S2). The confidence intervals for the estimation of the coefficients by 60000 bootstrap repetitions for the models in crystalized and fluid abilities that have as an only predictor network functional connectivity at DMN DAN and VAN. The dashed red line indicates the 25-percentile limit of the CI, the blue one the 95% percentile of the distribution. The lower panel shows the homologous distributions for the models in fluid intelligence. The purple lines or rug plots bellow the x-axis of all histograms represent the distribution of β coefficients estimation for every bootstrap repetition.*

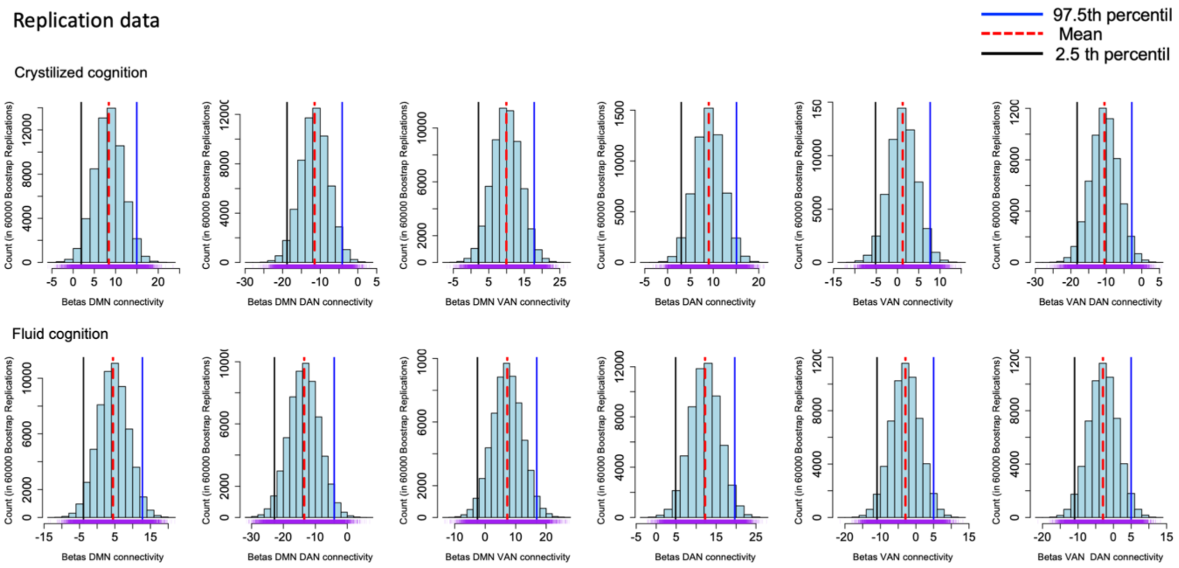

Figure S3 The Histograms shows the *distribution of β coefficients for the linear models in the replication data set. The figure shows the confidence intervals for the estimation of the coefficients by 60000 bootstrap repetitions for the models in crystalized and fluid abilities that have as an only predictor network functional connectivity at DMN DAN and VAN. The dashed red line indicates the 25-percentile limit of the CI, the blue one the 95% percentile of the distribution. The lower panel shows the homologous distributions for the models in fluid intelligence. The purple lines in the histogram represent the distribution of β coefficients.*

	Crystalized cognition	
	Confidence interval (***Discovery sample)***	
	2.5th	*97.5th*
DMN	-0.48827	12.66248
**DMN DAN**	***-20.38352***	***-6.104881***
DMN VAN	***4.559389***	***20.93797***
VAN	*-4.962465*	*7.46165*
**DAN**	***4.75945***	***15.8158***
**VAN DAN**	***-20.14513***	***-5.002945***

	Fluid cognition	
	Confidence interval (***Discovery sample)***	
	2.5th	*97.5th*
DMN	*-0.4332303*	*16.66822*
**DMN DAN**	***-25.53646***	***-7.137208***
DMN VAN	*0.1613086*	*20.40811*
VAN	*-5.182352*	*10.91381*
**DAN**	***6.58441***	***20.41042***
VAN DAN	*-23.00082*	*-4.126557*

	Crystalized cognition	
	Confidence interval (***Replication sample)***	
	2.5th	*97.5th*
DMN	-3.850174	*12.74695*
**DMN DAN**	***-22.86226***	***-4.023125***
DMN VAN	*-2.508508*	*16.90786*
VAN	*-10.96517*	*4.994669*
**DAN**	***4.829013***	***19.60904***
**VAN DAN**	***-18.19237***	***-2.825864***

	Fluid cognition	
	Confidence interval (***Replication sample)***	
	2.5th	*97.5th*
DMN	*-0.4332303*	*16.66822*
**DMN DAN**	***-25.53646***	***-7.137208***
DMN VAN	*0.1613086*	*20.40811*
VAN	*-5.182352*	*10.91381*
**DAN**	***6.58441***	***20.41042***
VAN DAN	*-16.63103*	*2.65191*

Table S2 *The table report the confidence interval for the estimation of the β estimates of the simplest linear models having as the only predictor network FC within and between network connectivity (same models than in Figures S3 and S4). The bootstrap estimate was calculated by 600000 repetitions for the models having as a predictor the composite score in fluid and crystallized cognition in the discovery (N=1353) and replication split (N=1354).*

	test (N=894)	train (N=1813)	P-value
**Age (months first session)**			
Mean (SD)	120 (7.65)	120 (7.57)	0.696
Median [Min, Max]	121 [107, 132]	120 [107, 132]	
**Age (months first session)**			
Boys	464 (51.9%)	838 (46.2%)	0.00613
Girls	430 (48.1%)	975 (53.8%)	
**Household Income (year) in USD**			
[<50K]	498 (55.7%)	1002 (55.3%)	0.93
[>=100K]	132 (14.8%)	263 (14.5%)	
[>=50K & <100K]	264 (29.5%)	548 (30.2%)	
**Highest-degreed parent (years)**			
< HS Diploma	34 (3.8%)	81 (4.5%)	0.452
Bachelor	345 (38.6%)	668 (36.8%)	
HS Diploma/GED	86 (9.6%)	163 (9.0%)	
Post Graduate Degree	289 (32.3%)	641 (35.4%)	
Some College	140 (15.7%)	260 (14.3%)	
**MRI manufacturer**			
GE MEDICAL SYSTEMS	254 (28.4%)	508 (28.0%)	0.867
SIEMENS	640 (71.6%)	1305 (72.0%)	
**Average framewise displacement in mm**			
Mean (SD)	0.105 (0.0436)	0.106 (0.0433)	0.812
Median [Min, Max]	0.0979 [0.0208, 0.199]	0.0990 [0.0196, 0.200]	
**NIH List Sorting Working Memory Score**			
Mean (SD)	98.7 (11.4)	99.1 (10.8)	0.388
Median [Min, Max]	97.0 [36.0, 136]	101 [47.0, 136]	
**Fluid intelligence Score NIH**			
Mean (SD)	94.3 (9.58)	94.2 (9.73)	0.835
Median [Min, Max]	95.0 [59.0, 122]	95.0 [59.0, 123]	
**Crystilized intelligence Score NIH**			
Mean (SD)	88.2 (6.67)	88.1 (6.86)	0.702
Median [Min, Max]	88.0 [59.0, 112]	88.0 [59.0, 115]	
**Picture Vocabulary Test Score NIH**			
Mean (SD)	86.5 (7.80)	86.2 (8.07)	0.49
Median [Min, Max]	86.0 [47.0, 111]	86.0 [36.0, 118]	
**Composite crystilized cognition**			
Mean (SD)	87.3 (7.04)	87.2 (7.28)	0.573
Median [Min, Max]	87.0 [53.0, 109]	86.5 [47.5, 117]	
**Composite fluid cognition**			
Mean (SD)	96.5 (9.38)	96.7 (9.20)	0.681
Median [Min, Max]	97.0 [55.0, 126]	97.0 [61.0, 129]	

Table S3 *The table reports the main features of the samples for cross validation either using kfolds and LASSO. accuracy performance of the models was computed using the training data (N=1813) and obtaining the predicted values in the test data. Prediction accuracy was measured comparing the predicted vs observed values of the models in the test data sample (N=894), correspond with results in Table 1.*

	discovery (N=1353)	replication (N=1354)	TRUE (N=2707)
**Sex**			
Boys	645 (47.7%)	657 (48.5%)	1302 (48.1%)
Girls	708 (52.3%)	697 (51.5%)	1405 (51.9%)
**Age**			
Mean (SD)	120 (7.61)	120 (7.57)	120 (7.59)
Median [Min, Max]	121 [107, 132]	120 [107, 132]	120 [107, 132]
**site_id_l**			
site02	112 (8.3%)	94 (6.9%)	206 (7.6%)
site03	73 (5.4%)	83 (6.1%)	156 (5.8%)
site04	114 (8.4%)	113 (8.3%)	227 (8.4%)
site05	35 (2.6%)	50 (3.7%)	85 (3.1%)
site06	104 (7.7%)	81 (6.0%)	185 (6.8%)
site07	35 (2.6%)	30 (2.2%)	65 (2.4%)
site08	46 (3.4%)	47 (3.5%)	93 (3.4%)
site09	57 (4.2%)	44 (3.2%)	101 (3.7%)
site10	83 (6.1%)	67 (4.9%)	150 (5.5%)
site11	49 (3.6%)	49 (3.6%)	98 (3.6%)
site12	67 (5.0%)	66 (4.9%)	133 (4.9%)
site13	80 (5.9%)	107 (7.9%)	187 (6.9%)
site14	67 (5.0%)	78 (5.8%)	145 (5.4%)
site15	36 (2.7%)	21 (1.6%)	57 (2.1%)
site16	206 (15.2%)	208 (15.4%)	414 (15.3%)
site18	41 (3.0%)	55 (4.1%)	96 (3.5%)
site20	76 (5.6%)	87 (6.4%)	163 (6.0%)
site21	67 (5.0%)	70 (5.2%)	137 (5.1%)
site22	5 (0.4%)	4 (0.3%)	9 (0.3%)
**Income**			
[<50K]	760 (56.2%)	740 (54.7%)	1500 (55.4%)
[>=100K]	193 (14.3%)	202 (14.9%)	395 (14.6%)
[>=50K & <100K]	400 (29.6%)	412 (30.4%)	812 (30.0%)
**education**			
< HS Diploma	68 (5.0%)	47 (3.5%)	115 (4.2%)
Bachelor	485 (35.8%)	528 (39.0%)	1013 (37.4%)
HS Diploma/GED	129 (9.5%)	120 (8.9%)	249 (9.2%)
Post Graduate Degree	464 (34.3%)	466 (34.4%)	930 (34.4%)
Some College	207 (15.3%)	193 (14.3%)	400 (14.8%)
**MRI_manufacturer**			
GE MEDICAL SYSTEMS	370 (27.3%)	392 (29.0%)	762 (28.1%)
SIEMENS	983 (72.7%)	962 (71.0%)	1945 (71.9%)
**FD**			
Mean (SD)	0.108 (0.0438)	0.104 (0.0429)	0.106 (0.0434)
Median [Min, Max]	0.100 [0.0196, 0.200]	0.0956 [0.0231, 0.200]	0.0988 [0.0196, 0.200]
**nihtbx_list_uncorrected**			
Mean (SD)	98.6 (11.0)	99.4 (10.9)	99.0 (11.0)
Median [Min, Max]	97.0 [36.0, 128]	101 [55.0, 136]	101 [36.0, 136]
**nihtbx_fluidcomp_uncorrected**			
Mean (SD)	94.0 (9.77)	94.4 (9.58)	94.2 (9.68)
Median [Min, Max]	94.0 [59.0, 123]	95.0 [59.0, 122]	95.0 [59.0, 123]
**nihtbx_cryst_uncorrected**			
Mean (SD)	87.9 (6.86)	88.3 (6.73)	88.1 (6.80)
Median [Min, Max]	87.0 [59.0, 115]	88.0 [64.0, 115]	88.0 [59.0, 115]
**nihtbx_picvocab_uncorrected**			
Mean (SD)	86.1 (8.07)	86.5 (7.89)	86.3 (7.98)
Median [Min, Max]	85.0 [36.0, 118]	86.0 [59.0, 115]	86.0 [36.0, 118]
**composite_crystilized**			
Mean (SD)	87.0 (7.28)	87.4 (7.12)	87.2 (7.20)
Median [Min, Max]	86.5 [47.5, 117]	87.0 [64.5, 114]	86.5 [47.5, 117]
**composite_fluid**			
Mean (SD)	96.3 (9.37)	96.9 (9.14)	96.6 (9.26)
Median [Min, Max]	97.0 [55.0, 121]	97.5 [61.5, 129]	97.0 [55.0, 129]

Table S4 *The table reports the main features of the samples for internal validation with half-split calculation of β coefficient and confidence intervals of the regression models by 600000 bootstrap repetitions in the discovery (N=1353) and replication samples (N=1354)*

	nihtbx_cryst_uncorrected	nihtbx_cryst_uncorrected_PPVT	nihtbx_fluidcomp_uncorrected	nihtbx_fluidcomp_uncorrected_ListSorting
Age	0.0000000	0.0000000	0.0000000	0.000000
SexGirls	0.0000000	0.0000000	0.0000000	0.000000
Income[>=50K & <100K]	0.0000000	0.0000000	0.0000000	0.000000
Income[>=100K]	0.0376304	0.0000000	0.0000000	0.000000
site_id_lsite03	0.0000000	0.0000000	0.0000000	0.000000
site_id_lsite04	0.0000000	0.0000000	0.0000000	0.000000
site_id_lsite05	0.0000000	0.0000000	0.0000000	0.000000
site_id_lsite06	0.0000000	0.0000000	0.0000000	0.000000
site_id_lsite07	0.0000000	0.0000000	0.0000000	0.000000
site_id_lsite08	0.0000000	0.0000000	0.0000000	0.000000
site_id_lsite09	0.0000000	0.0000000	0.0000000	0.000000
site_id_lsite10	0.0000000	0.0000000	0.0000000	0.000000
site_id_lsite11	0.0000000	0.0000000	0.0000000	0.000000
site_id_lsite12	0.0000000	0.0000000	0.0000000	0.000000
site_id_lsite13	0.0000000	0.0000000	0.0000000	0.000000
site_id_lsite14	0.0000000	0.0000000	0.0000000	0.000000
site_id_lsite15	0.0000000	0.0000000	0.0000000	0.000000
site_id_lsite16	0.0000000	0.0000000	0.0000000	0.000000
site_id_lsite18	0.0000000	0.0000000	0.0000000	0.000000
site_id_lsite20	0.0000000	0.0000000	0.0000000	0.000000
site_id_lsite21	0.0000000	0.0000000	0.0000000	0.000000
site_id_lsite22	0.0000000	0.0000000	0.0000000	0.000000
educationHS Diploma/GED	-2.3324306	-2.4953376	-2.4539346	0.000000
educationSome College	-0.2745195	-0.2321583	0.0000000	0.000000
educationBachelor	1.0127493	0.3599866	0.0000000	0.000000
educationPost Graduate Degree	2.4863864	1.8891803	0.8049197	0.000000
FD	-1.0218409	0.0000000	-6.7137080	-3.270310
DMN	0.0000000	0.0000000	0.0000000	0.000000
DAN	5.0126301	4.7455868	8.6436181	4.094882
VAN	0.0000000	0.0000000	0.0000000	0.000000
DMN_DAN	-0.6322184	0.0000000	0.0000000	0.000000
DMN_VAN	3.1926722	2.7451322	0.0000000	0.000000
VAN_DAN	0.0000000	0.0000000	0.0000000	0.000000
FPN	0.0000000	0.0000000	5.8901382	0.000000
FPN_DAN	5.0682434	4.1644100	0.0000000	0.000000
FPN_VAN	0.0000000	0.0000000	0.0000000	0.000000
DMN_FPN	0.0000000	0.0000000	0.0000000	0.000000

Table S5. *The table reports the results of the LASSO models in the whole population (N=2707) having in account within-network connectivity of the frontoparietal network (FPN), and the between network connectivity of the FPN with the default mode network (DMN) and attentional networks (VAN and DAN). Every column of the table is a multivariate regression model, and rows predictors. There are four models: the Crystallized Composite Uncorrected Standard Score ("nihtbx_cryst_uncorrected"), the average of the Crystallized Composite score and the uncorrected Standard Score and NIH Toolbox Picture Vocabulary test ("nihtbx_picvocab_uncorrected"), or our composite score in crystallized abilities. The third column reports the β coefficients of the fluid cognition composite uncorrected standard score ("nihtbx_fluidcomp_uncorrected"), and fourth column the average of List Sorting Working Memory Score and ("nihtbx_list_uncorrected") and the fluid cognition composite uncorrected standard score, or our composite score on fluid abilities. The table reports the β coefficients of the cross-validated LASSO regression obtained al λ.1se. It can be observed that most of the coefficients for all models shrink to zero. Within-network connectivity of the DAN appears as a common factor explaining fluid and crystallized abilities while between-network connectivity of the DMN and attentional networks appears as the more relevant feature explaining crystallized abilities. These results are independent of the use of raw score of the NIH toolbox on cristilized and fluid intelligence or the composite scores in crystalized and fluid abilities. Importantly, even when the whiting-network connectivity of the FPN appears as a significant feature associated with fluid abilities, these predictions were not confirmed by the partial correlations analysis reported in Table S5 between functional connectivity of every resting-state network and the cognitive outcome. FPN (withing network connectivity FPN), FPN DAN (between-network connectivity FPN DAN), FPN DAN (between-network connectivity FPN VAN), and DMN FPN (between-network connectivity FPN DMN).*

	nihtbx_cryst_uncorrected_PPVT			nihtbx_cryst_uncorrected			nihtbx_fluidcomp_uncorrected_ListSorting			nihtbx_fluidcomp_uncorrected		
	ρ	***t***	***p***	*ρ*	***t***	***p***	***rho***	***t***	***p***	*ρ*	***t***	***p***
DMN	.05	3.0	> 0.5	.05 **	2.9	> 0.5	.01	0.63	> 0.5	.01	.76	> 0.5
VAN	.01	0.7	> 0.5	0.01	0.8	> 0.5	-.01	-0.4	> 0.5	.006	.32	> 0.5
DAN	**.08** (**)	4.6	.001	**.08** (*)	4.2	.012	**.08** (*)	4.2	.013	**.08** (**)	4.18	.014
FPN	-.006	-0.3	> 0.5	.00	0.0	> 0.5	.04 *	2.1	> 0.5	.03	1.86	> 0.5
DMN_DAN	**-.08** (**)	-4.4	.004	**-.07** (*)	4.1	.014	-.05 **	-2.7	> 0.5	-.05 **	-3.0	> 0.5
DMN_VAN	**.08** (*)	4.2	.01	.07 ***	3.6	> 0.5	.02	1.3	> 0.5	.02	1.50	> 0.5
VAN_DAN	-.06 **	-3.6	> 0.5	-.06 ***	-3.3	> 0.5	-.04 *	-2.1	> 0.5	-.04 *	-2.3	> 0.5
FPN_DAN	.01	0.8	> 0.5	.02	1.1	> 0.5	-.01	-0.9	> 0.5	.00	-0.2	> 0.5
FPN_VAN	.01	0.7	> 0.5	.01	0.6	> 0.5	.01	0.8	> 0.5	.01	0.84	> 0.5
DMN_FPN	.001	0.07	> 0.5	.006	0.3	> 0.5	.00	0.1	> 0.5	-.01	-0.7	> 0.5

Table S6 *The table reports the Spearman partial correlation coefficients ρ in the whole cohort (N=2707) between the cognitive outcomes on crystallized and fluid abilities with functional connectivity of the resting-state networks in the whole population. The* ***t*** *is the t-statistics estimate, ρ the Spearman rho for the correlation between functional connectivity and cognition controlled by age and head movement in the scan (FD). p are Bonferroni corrected p-values.* The symbol **(*)** denotes significant after Bonferroni correction, and ***** significant without multiple comparison correction.

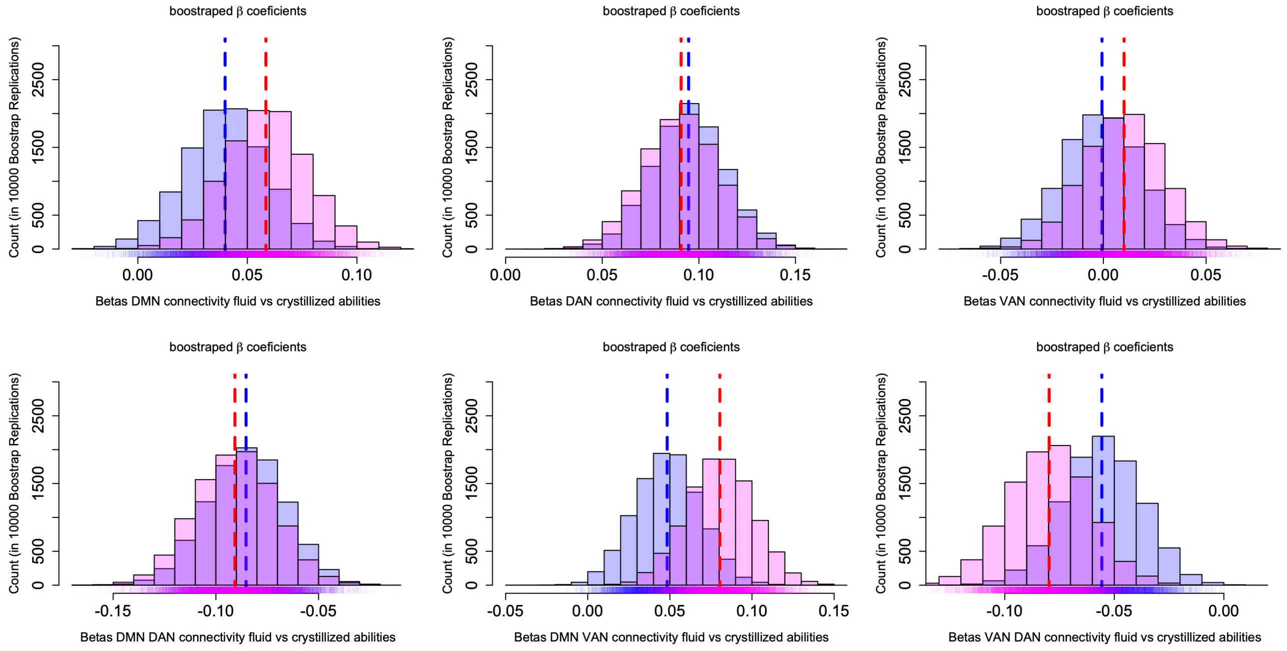

Figure S4 The figure shows the comparison of bootstrap distributions from 10000 repetitions of standardized *β* coefficients *βs= βr (σx / σy)* for the models having as an outcome the average of the crystallized intelligence score and picture vocabulary (red), and fluid abilities measured as the average of fluid cognition score and list shorting memory task (in blue) in the whole sample (N=2707). The histograms show the trend: more negative (anticorrelated) values of DMN DAN (two-samples Kolmorogov-Smirnov Distance D=0.136,p<0.001), and VAN DAN (two-samples Kolmorogov-Smirnov Distance D=0.5729,p<0.001) predict positive changes in crystallized cognition, and more positive (correlated) values of DMN VAN connectivity predicts higher scores in crystallized abilities compared with the average estimate of the *β* coefficients of fluid skills (dashed line in blue average of the bootstrap distribution for fluid capacities, and red for crystallized skills). On the other hand, note that the mean weight *β* coefficients of the DAN lie above the distributions β coefficients having as an outcome fluid ability (two-samples Kolmorogov-Smirnov Distance D=0.0899, p<0.001), suggesting that more local functional connectivity changes within the frontoparietal nodes of the DAN are more important for the emergence of fluid abilities than for crystallized skills. Individual estimations of standardized β coefficients for every replicate are represented in the superimposed rug plots bellow the x-axis for both models (red= crystallized abilities, and bleu= fluid abilities).
